# Efficacy and safety of primary posterior capsulotomy during phaco-vitrectomy for epiretinal membrane

**DOI:** 10.1186/s12886-021-02226-5

**Published:** 2022-01-03

**Authors:** Ki Won Jin, Se Joon Woo, Kyu Hyung Park

**Affiliations:** Department of Ophthalmology, Seoul National University College of Medicine, Seoul National University Bundang Hospital, 173-82 Gumi-ro, Bundang-gu, Seongnam-si, Gyeonggi-do 13620 South Korea

**Keywords:** Posterior capsulotomy, Phaco-vitrectomy, Idiopathic epiretinal membrane, Posterior capsular opacity

## Abstract

**Purpose:**

To evaluate the necessity and safety of primary posterior capsulotomy during phaco-vitrectomy for idiopathic epiretinal membrane (ERM).

**Setting:**

Seoul National University Bundang Hospital, Seongnam, Korea.

**Design:**

Retrospective consecutive cohort analysis.

**Methods:**

This study enrolled 219 patients (228 eyes) who underwent combined 25-gauge phaco-vitrectomy for idiopathic ERM and cataract, divided into capsulotomy (−) group (152 eyes, 144 patients) and capsulotomy (+) group (76 eyes, 75 patients). The main outcomes were rate of posterior capsular opacity (PCO) occurrence and postoperative complications. Ophthalmic examinations were performed at baseline, 1, 3, 6, and 12 months postoperatively.

**Results:**

PCO only occurred in capsulotomy (−) group (20 eyes, 13.2%), with mean onset of 10.59 months. Visually-significant PCO that needed Nd:YAG posterior capsulotomy was present in 9 eyes (45.0% of PCO eyes). The rate of cystoid macular edema (CME) was higher in capsulotomy (+) group (6.6% vs. 15.8%, *p* = 0.026) with longer duration (1.50 vs. 3.36 months, *p* = 0.019). Female sex and posterior capsulotomy were significant risk factors for CME occurrence (*p* < 0.05).

**Conclusion:**

Primary posterior capsulotomy during phaco-vitrectomy for idiopathic ERM obviated the need for Nd:YAG posterior capsulotomy, but visually-significant PCO that needed Nd:YAG laser was not common. Considering the low rate of visually-significant PCO and high rate of postoperative CME, routine posterior capsulotomy during phaco-vitrectomy may not be necessary for preventing PCO in ERM.

## Introduction

Epiretinal membrane (ERM) is a common retinal disorder in older individuals characterized by fibroglial cellular proliferation on the inner surface of macula [1]. The formation of ERM formation is regulated by extracellular matrix, cytokines, growth factors, and miRNAs [2, 3]. The contractile membrane can cause distortion of fovea, thus leads to decreased and distorted vision [4]. Surgical treatment of membrane peeling shows improved visual acuity and metamorphopsia [5–7].

The combined surgery of pars plana vitrectomy (PPV) with phacoemulsification and intraocular lens (IOL) implantation is widely used for various vitreoretinal diseases, including ERM, co-existing with cataract [8, 9]. The phaco-vitrectomy procedure allows cost-effectivity and faster recovery, [10] with low complication rate [11]. However, previous studies have shown a possible occurrence of posterior capsular opacity (PCO) as the most common postoperative complication [12, 13].

Gimbel in 1990 first suggested posterior continuous curvilinear capsulorrhexis (PCCC) for posterior capsule opacities or tears during adult cataract extraction [14]. It involves removal of the central part of the posterior capsule to prevent equatorial lens epithelial cell (LEC) migration and formation of PCO, and has been utilized as a primary planned procedure for the prevention of PCO formation in adults [15, 16]. The PCCC procedure eradicates the need for Neodymium:yttrium aluminum garnet (Nd:YAG) laser capsulotomy, that is associated with numerous complications including retinal tears, IOL damage, cystoid macular edema (CME), anterior hyaloid opacity, inflammation, and increased intraocular pressure (IOP) [17–19].

Recently, posterior capsulotomy during phaco-vitrectomy using a vitreous cutter, employing the pars plana approach, was reported to be a simple procedure without a steep learning curve, unlike PCCC [20–22]. Previous studies have shown excellent efficacy of posterior capsulotomy, but the subjects either had heterogenous retinal disease [20, 21] or rhegmatogenous retinal detachment with a high risk of postoperative PCO [22]. Additionally, in the pseudophakic cases with preoperative PCO, primary posterior capsulotomy can be adopted to enhance the intraoperative visualization of the retina. However, studies evaluating posterior capsulotomy during macular surgery are lacking. Furthermore, studies reporting the safety of posterior capsulotomy are scarce in the literature, [20, 22] thereby necessitating quantitative evaluation of the safety of posterior capsulotomy.

The purpose of the present study was to evaluate the efficacy and safety of primary posterior capsulotomy during 25-gauge phaco-vitrectomy for idiopathic epiretinal membrane (ERM).

## Materials and methods

### Design and patients

In this retrospective cohort study, the medical records of all consecutive patients who visited the retina clinic at the Department of Ophthalmology of Seoul National University Bundang Hospital with idiopathic ERM and co-existing cataract, and underwent combined 25-gauge phaco-vitrectomy between January 2015 and June 2019 were reviewed. Only patients followed-up for at least 6 months after the surgery were included. The following were the exclusion criteria: secondary ERM, combined phaco-vitrectomy for co-existing macular disease other than idiopathic ERM, including macular hole, vitreomacular traction, and myopic tractional maculopathy, previous intraocular surgery, any complications during cataract extraction, utilization of intraocular tamponade during surgery, and re-operation during study period.

### Surgical technique and patient grouping

Two expert surgeons (K.H.P., S.J.W.) performed the surgeries, and only S.J.W. conducted primary posterior capsulotomy according his preference. Cataract extraction was performed by phacoemulsification with posterior chamber IOL implantation via a clear corneal incision, followed by a 25-gauge PPV with the Constellation Vision System (Alcon Laboratories, Inc., Fort Worth, TX). Posterior vitreous detachment was induced by active suction using an ocutome, if not already present. Complete removal of the posterior hyaloid was done. The ERM and internal limiting membrane (ILM) were peeled with microforceps. Of note, the ILM peeling was assisted with the use of triamcinolone (TA) (MaQaid, Wakamoto Pharmaceutical Co., Ltd., Tokyo, Japan) in cases performed by the surgeon K.H.P., or indocyanine green (ICG) (Diagnogreen, Daiichi-Sankyo Pharma, Tokyo, Japan) 0.125% (1.25 mg/mL) in cases performed by the other surgeon, S.J.W. After the membrane peeling, limited partial peripheral vitrectomy was performed without vitreous base shaving. None of the cases utilized intraocular tamponade agents or intraocular injections.

**Fig. 1:**
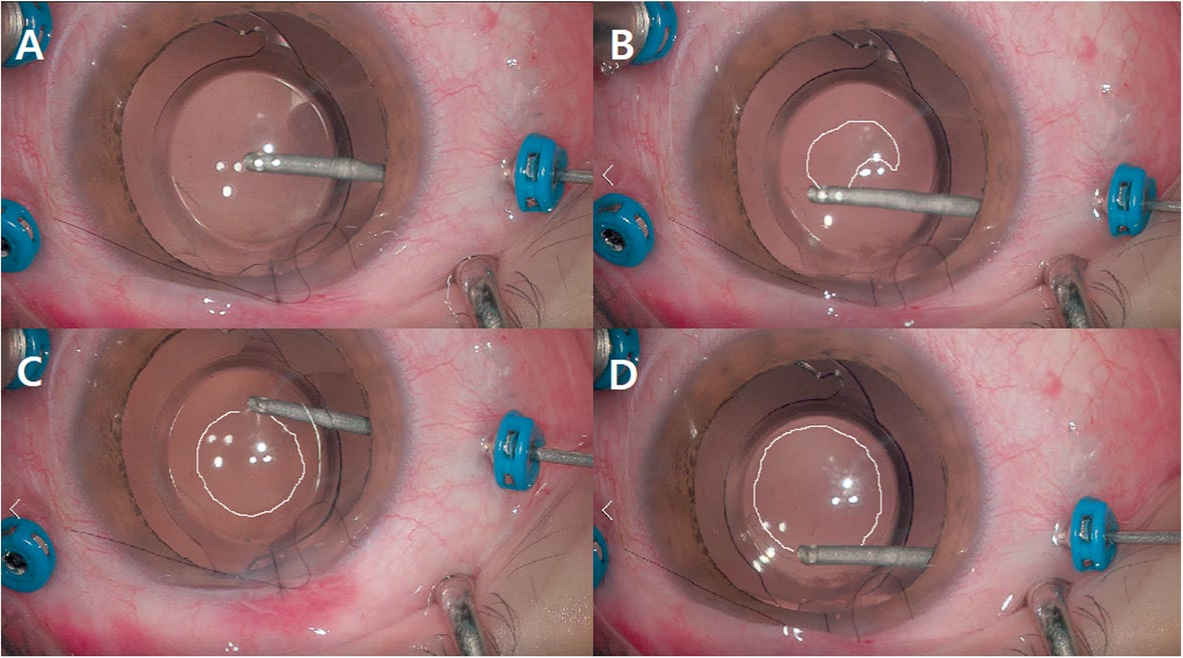
Primary posterior capsulotomy was performed in a sequential manner (**A** to **D**) during phaco-vitrectomy. Consequently, a 4–5 mm wide, well-centered posterior capsulotomy site was created

At the end of the surgery, one surgeon (S.J.W.) removed the posterior capsule using a vitreous cutter by the pars plana approach. A well-centered posterior capsulotomy measuring 4–5 mm in diameter was made in a centrifugal, circular manner (Fig. 1). The patients were grouped into capsulotomy (−) group and capsulotomy (+) groups. Trocars were removed afterward. Postoperative intravenous NSAIDS was given when needed. Patients were treated postoperatively with topical levofloxacin 0.5% 4 times a day, topical prednisolone 1% 4 times a day and maintained the eye drops for 1 month.

### Patient evaluation

**Fig. 2:**
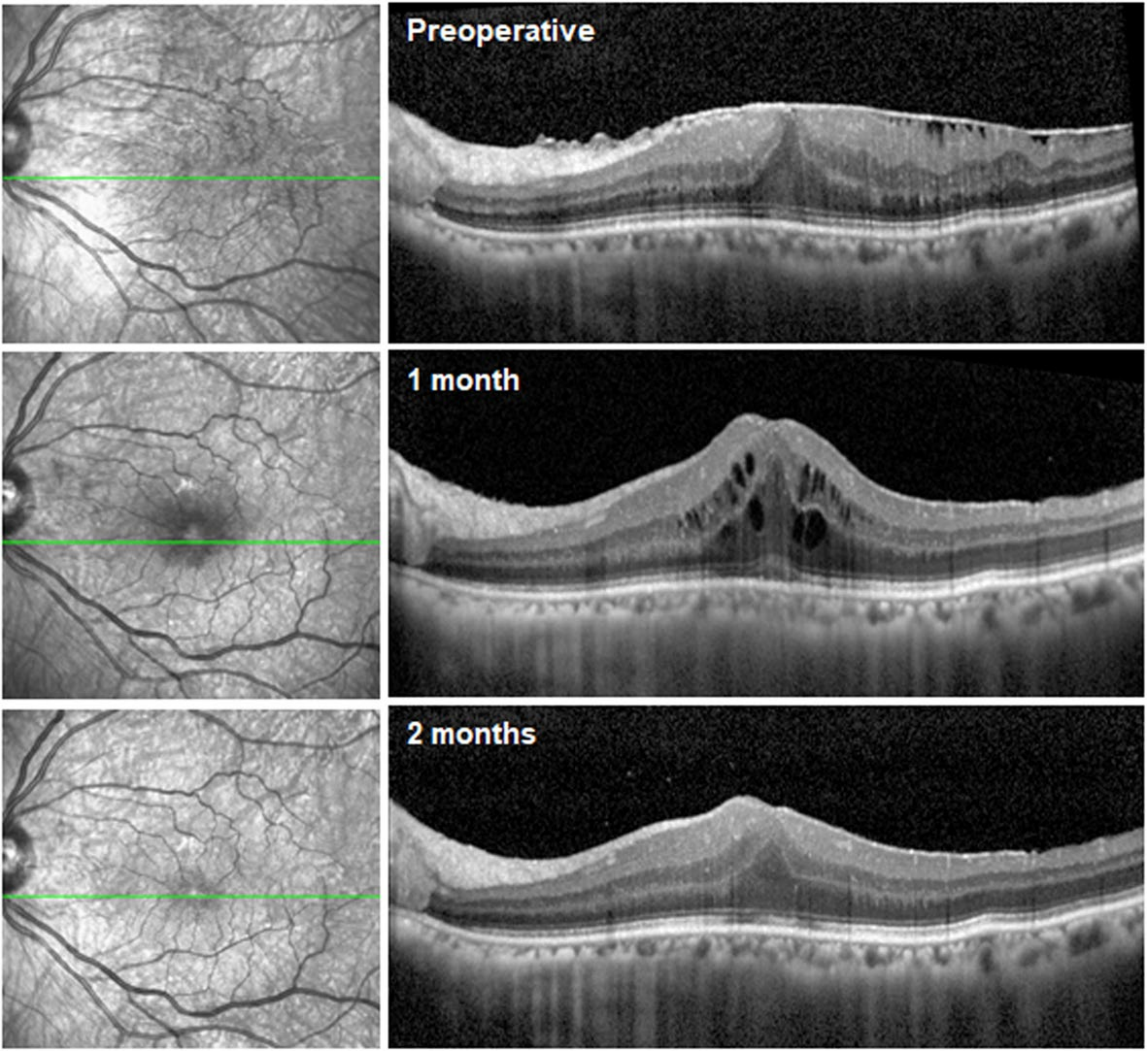
Representative case of postoperative cystoid macular edema occurrence after the epiretinal membrane surgery. The perioperative and postoperative horizontal scan optical coherent tomography images (left column) and infrared imaging (right column) centered at the fovea was presented. A 59-year-old female patient received epiretinal membrane removal with primary posterior capsulotomy. After 1 month postoperatively, cystoid macular edema occurred and the patient was treated with topical bromfenac 0.1% twice a day. After 1 month of the treatment, the macular edema was resolved

We performed baseline preoperative examinations including the slit-lamp examination, fundoscopy, best-corrected visual acuity (BCVA) measured in decimals, IOP measurement by a noncontact tonometer (KT-800; KOWA, Tokyo, Japan), color fundus photography (VX-10a; KOWA, Tokyo, Japan), and optical coherence tomography (OCT, Spectralis; Heidelberg Engineering, Heidelberg, Germany). After undergoing phaco-vitrectomy, the patients visited the clinic at 1, 3, 6, and 12 months postoperatively. At each visit, complete ophthalmic examinations as those done at baseline were performed. The decimal visual acuities were converted into the logarithm of the minimum angle of resolution (LogMAR) unit for arithmetical comparisons. The extent of preoperative ERM was staged using the staging scheme proposed by Govetto et al., based on OCT findings [23]. PCO occurrence was checked by slit-lamp examination, and Nd:YAG posterior capsulotomy was performed using an ophthalmic YAG photodisruptor laser (Aura PT; Lumenis, Inc., Santa Clara, CA). The occurrence of postoperative CME was evaluated by OCT at each visit. CME was defined as newly presented cystic spaces in the outer plexiform and inner nuclear layer of the retina on postoperative OCT. Representative case of postoperative CME were presented (Fig. 2). Central macular thickness (CMT) was defined as the thickness between inner surface of retina and the outer boundary of the retinal pigment epithelium at the fovea, and was measured manually using the calipers provided by the Spectralis Heidelberg software during the horizontal macular scan of the foveal center. ERM recurrence was defined as recurrence of the membrane that had been removed at surgery or regrowth of the residual ERM, and evaluated by postoperative fundus examination or fundus photography.

### Outcome measures

The main outcomes were the rate of posterior capsular opacity (PCO) occurrence and postoperative complications, including postoperative inflammation until 3 months, corneal edema, IOP increase above 30 mmHg at 1 month, IOL-related complications, anterior capsular phimosis, CME, postoperative retinal breaks, and ERM recurrence. Visual and structural surgical outcomes between groups were compared in the forms of postoperative BCVA and CMT changes from baseline measurements.

### Statistical analysis

All analyses were performed using a statistical software, SPSS for Windows Version 25.0 (IBM Corp., Armonk, NY, USA). A *p*-value less than 0.05 was considered statistically significant. The chi-square test was conducted to compare categorical variables between groups. Continuous variables including BCVA change and CMT change from baseline were compared with independent t-tests. The correlation between CME and other postoperative complications were evaluated by Spearman correlation analysis. Multivariate logistic regression analysis was employed to determine the associations of the demographic, preoperative, and operative variables with the occurrence of CME.

## Results

A total of 347 consecutive eyes of 335 patients who underwent combined 25-gauge phaco-vitrectomy for ERM and co-existing cataract were identified. From this cohort, patients were excluded according to the exclusion criteria as follows: secondary ERM (34 eyes of 33 patients), combined phaco-vitrectomy for other co-existing maculopathy than ERM (14 eyes of 14 patients), utilization of intraocular tamponade during surgery (4 eyes in 4 patients; 1 intravitreal gas injection, 3 air injections), re-operation within the study period (1 eye with macular hole occurrence), and lost to follow-up within 6 months (66 eyes of 64 patients). Consequently, 152 eyes of 144 subjects in the capsulotomy (−) group and 76 eyes of 75 subjects in the capsulotomy (+) group were finally included in the analysis.

The demographic characteristics of subjects and preoperative ERM evaluation were compiled in Table 1. The mean age of the subjects was 67.45 ± 6.60 years in the capsulotomy (−) group, and 65.68 ± 6.95 years in the capsulotomy (+) group (*p* = 0.062, independent t-test). Female patients were more frequent in the capsulotomy (−) group than capsulotomy (+) group (71.7% in capsulotomy (−) group vs 57.9% in capsulotomy (+) group, *p* = 0.036, pearson chi-square test). The mean follow-up duration was 11.41 ± 1.80 months in capsulotomy (−) group and 10.97 ± 2.27 months in capsulotomy (+) group (*p* = 0.118, independent t-test). The ERM stage did not differ between two groups (*p* = 0.124, independent t-test), but baseline CMT was significantly thicker in the capsulotomy (+) group (*p* = 0.000, independent t-test). Baseline visual acuities (logMAR) were not different between the groups (*p* = 0.233, independent t-test).

**Table 1:** Demographic and preoperative characteristics of patients

Characteristics	Capsulotomy (−) group (***n*** = 152)	Capsulotomy (+) group (***n*** = 76)	***P*** value
**Age (years), mean ± SD**	67.45 ± 6.60	65.68 ± 6.95	0.062*
**Sex (female), n (%)**	109 (71.7%)	44 (57.9%)	**0.036**^†^
**DM, n (%)**	20 (13.2%)	11 (14.5%)	0.785^†^
**HTN, n (%)**	58 (38.2%)	25 (32.9%)	0.436^†^
**Follow-up duration (months), mean ± SD**	11.41 ± 1.80	10.97 ± 2.27	0.118*
**ERM stage, n (%)**			0.124^†^
**Stage 1**	34 (22.4%)	9 (11.8%)	
**Stage 2**	32 (21.1%)	12 (15.8%)	
**Stage 3**	60 (39.5%)	38 (50.0%)	
**Stage 4**	26 (17.1%)	17 (22.4%)	
**Baseline BCVA (logMAR), mean ± SD**	0.308 ± 0.204	0.344 ± 0.229	0.233*
**Baseline CMT (μm), mean ± SD**	412.75 ± 112.48	483.37 ± 100.59	0.000*

*DM* Diabetes mellitus, *HTN* Hypertension, *ERM* Epiretinal membrane, *BCVA* Best-corrected visual acuity, *logMAR* Logarithm of the minimum angle of resolution, *CMT* Central macular thickness

*: Independent t-test, †: Pearson chi-square test

### Postoperative incidence of posterior capsular opacity

**Fig. 3:**
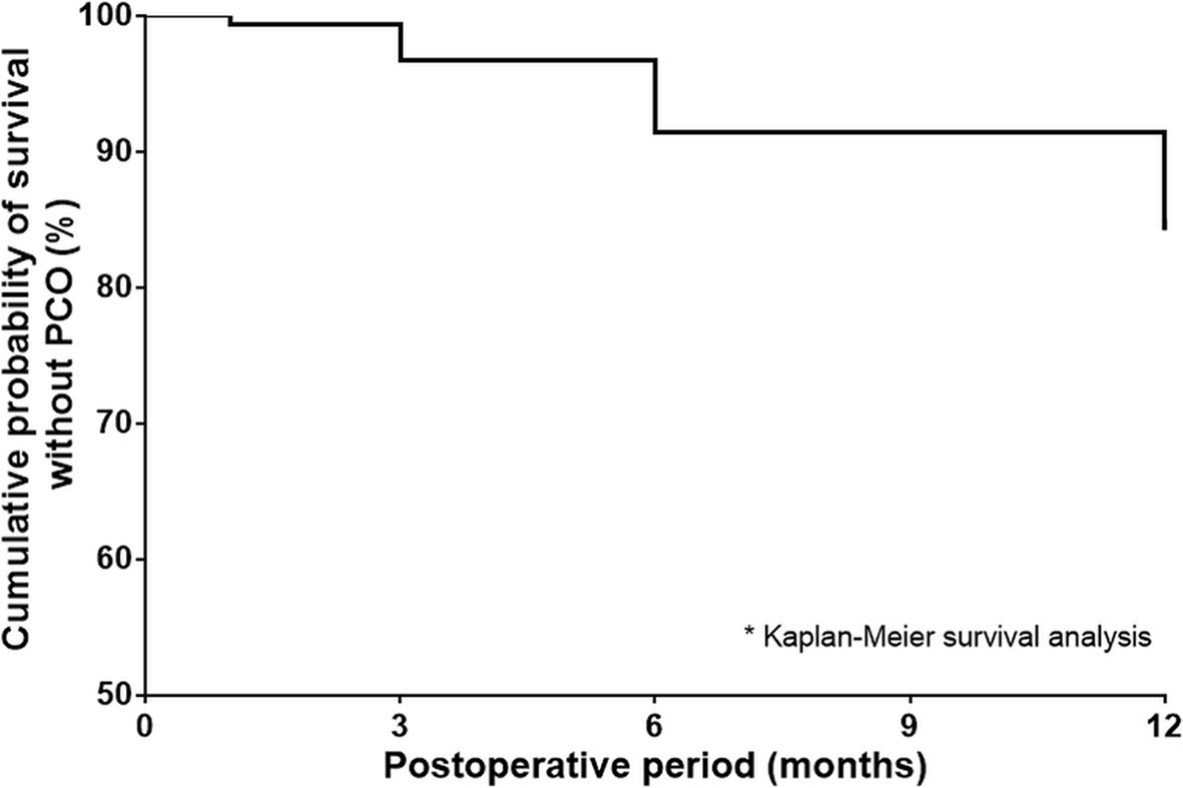
Survival analysis of posterior capsular opacity occurrence in the capsulotomy (−) group. PCO = posterior capsular opacity. * = Kaplan-Meier survival analysis

PCO was only reported in the capsulotomy (−) group (20 eyes, 13.2%, *p* = 0.001, pearson chi-square test). The mean onset of PCO was 10.59 ± 2.88 months, and visually-significant PCO needing Nd:YAG posterior capsulotomy was found in 9 eyes (45.0% of PCO eyes, 5.9% of total eyes) (Table 2). The Kaplan-Meier survival curve was plotted to show the cumulative probability of survival without PCO occurrence in the capsulotomy (−) group (Fig. 3).

**Table 2:** Posterior capsular opacity and postoperative complications after phaco-vitrectomy for Idiopathic epiretinal membrane

	Capsulotomy (−) group (***n*** = 152)	Capsulotomy (+) group (***n*** = 76)	***P*** value
**PCO, n (%)**	20 (13.2%)	0	0.001*
**Onset (months), mean ± SD**	10.59 ± 2.88	0	
**Nd:YAG posterior capsulotomy, n (%)**	9 (45.0%)	0	
**Anterior chamber inflammation, n (%)**	4 (2.6%)	1 (1.3%)	0.522*
**Corneal edema, n (%)**	2 (1.3%)	3 (3.9%)	0.201*
**IOP increase, n (%)**	1 (0.7%)	0	0.479*
**IOL decenter, tilt or dislocation, n (%)**	0	0	
**Anterior capsular phimosis, n (%)**	1 (0.7%)	2 (2.6%)	0.218*
**CME, n (%)**	10 (6.6%)	12 (15.8%)	0.026*
**Onset (months), mean ± SD**	1 ± 0	1.17 ± 0.57	0.374^†^
**Duration (months), mean ± SD**	1.50 ± 0.71	3.36 ± 2.20	**0.019**^†^
**Treatment, n (%)**			0.130*
**Observation**	0	3 (25.0%)	
**Topical eye drops**	10 (100%)	8 (66.7%)	
**Intravitreal TA injection**	0	1 (8.3%)	
**Postoperative retinal breaks, n (%)**	2 (1.3%)	0	0.315*
**ERM recurrence, n (%)**	26 (17.1%)	12 (15.8%)	0.802*
**Onset (months), mean ± SD**	5.81 ± 4.30	6.67 ± 4.33	0.571^†^

*PCO* Posterior capsular opacity, *Nd:YAG* Neodymium:yttrium aluminum garnet, *IOP* Intraocular pressure, *IOL* Intraocular lens, *CME* Cystoid macular edema, *TA* Triamcinolone, *ERM* Epiretinal membrane

*: Pearson chi-square test, †: Independent t-test

### Postoperative complications

The postoperative complications seen in both groups are presented in Table 2. The incidence of postoperative inflammation until 3 months, and transient corneal edema and postoperative IOP increase at 1 month did not differ significantly between the groups (*p* > 0.05, pearson chi-square test). IOL-related complications such as IOL decentration, IOL tilt, and dislocation did not occur. Anterior capsular phimosis was noted in 1 eye in the capsulotomy (−) group (0.7%) and 2 eyes in the capsulotomy (+) group (2.6%) (*p* = 0.218, pearson chi-square test), both whom underwent Nd:YAG anterior capsular contracture lysis. The postoperative incidence of CME was significantly higher in the capsulotomy (+) group than in the capsulotomy (−) group (6.6% in capsulotomy (−) group vs 15.8% in capsulotomy (+) group, *p* = 0.026, pearson chi-square test). While the mean onset of CME was not different between the groups (*p* = 0.374), the duration of CME was significantly longer in the capsulotomy (+) group (3.36 ± 2.20 months) than in the capsulotomy (−) group (1.50 ± 0.71 months) (*p* = 0.019, independent t-test). The treatment for CME in each group was not significantly different between groups. For eye drops, topical NSAIDS and/or steroid were prescribed, which include bromfenac 0.1%, fluorometholone 0.1%, loteprednol 0.5%, and prednisolone 0.1%. Incidences of postoperative peripheral retinal breaks and ERM recurrence were not different between the groups (*p* > 0.05, pearson chi-square test). Mild vitreous hemorrhage was noted in 1 eye at 1 month postoperatively in the capsulotomy (+) group.

We evaluated the risk factors for postoperative CME occurrence with multivariate logistic regression analysis (Table 3). Female sex (Odds ratio [OR] 4.177, 95% confidence interval [CI] 1.167–14.945, *p* = 0.028, multivariate logistic regression) and posterior capsulotomy during phaco-vitrectomy (OR 3.200, 95% CI 1.284–7.973, *p* = 0.013, multivariate logistic regression) were found to be independent risk factors for CME occurrence after ERM surgery.

**Table 3:** Multivariate logistic regression analysis of factors associated with incidence of cystoid macular edema after epiretinal membrane surgery

Factor	Odds ratio	95% Confidence Interval	***P*** value
**Age (years)**	0.986	(0.911, 1.065)	0.402
**Sex (female)**	4.177	(1.167, 14.945)	**0.028**
**DM**	0.880	(0.168, 4.611)	0.656
**HTN**	0.909	(0.325, 2.545)	0.675
**ERM stage**			0.505
**Stage I (reference)**	1.000		
**Stage II**	6.741	(0.785, 57.895)	0.595
**Stage III**	5.436	(0.616, 47.959)	0.752
**Stage IV**	14.443	(1.039, 200.855)	0.296
**Baseline BCVA (logMAR)**	0.343	(0.028, 4.170)	0.408
**Baseline CMT (μm)**	0.995	(0.988, 1.002)	0.964
**Posterior capsulotomy**	3.200	(1.284, 7.973)	**0.013**

*DM* Diabetes mellitus, *HTN* Hypertension, *ERM* Epiretinal membrane, *BCVA* Best-corrected visual acuity, *logMAR* Logarithm of the minimum angle of resolution, *CMT* Central macular thickness

### Changes of BCVA and CMT from baseline measures

**Fig. 4:**
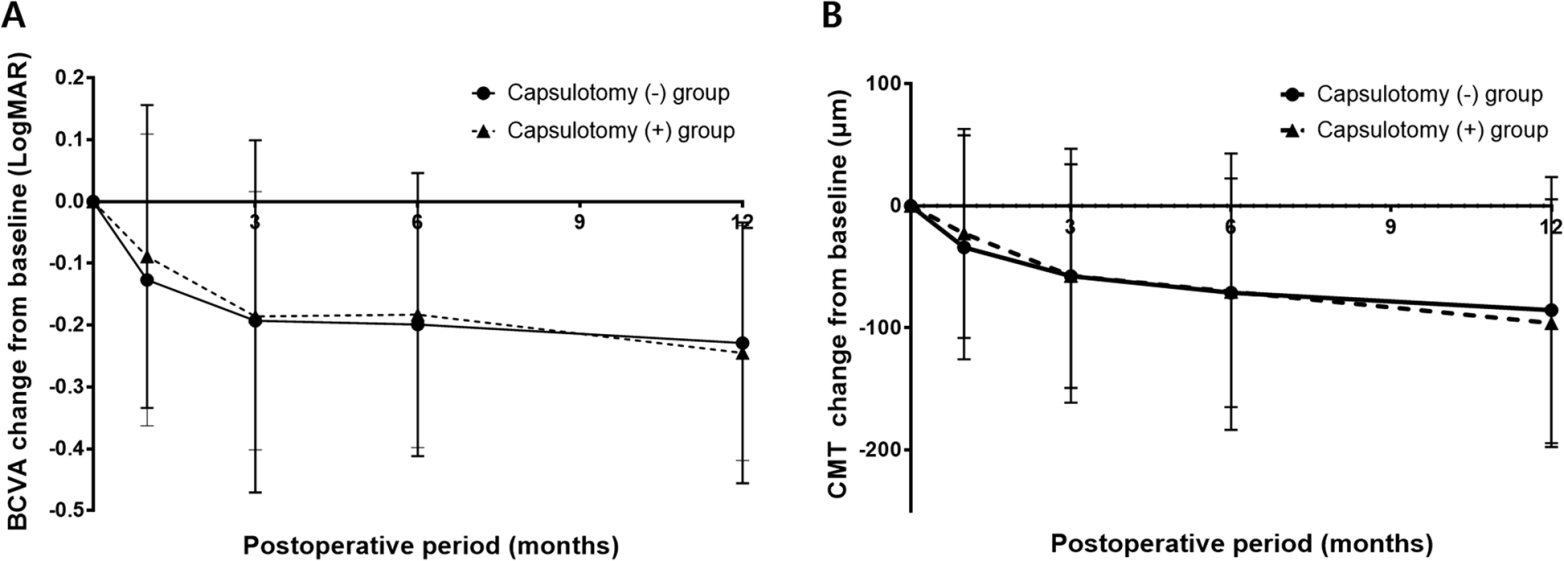
**A**. Postoperative changes in the best-corrected visual acuity from baseline. **B** Postoperative changes in the central macular thickness from baseline

Both groups showed better postoperative visual acuities and thinner CMT compared to baseline VA and CMT (*p* < 0.05, independent t-test) (Table 4). BCVA and CMT change from baseline at all postoperative follow-up periods were not significantly different between the groups (*p* > 0.05, independent t-test, Fig. 4).

**Table 4:** Postoperative changes of best-corrected visual acuity and central macular thickness from baseline evaluation

	Capsulotomy (−) group (***n*** = 152)	Capsulotomy (+) group (***n*** = 76)	***P*** value*
**BCVA change from baseline**			
**1 month, mean ± SD**	− 0.127 ± 0.236 (n = 152)	− 0.089 ± 0.245 (*n* = 76)	0.266
**3 months, mean ± SD**	− 0.193 ± 0.209 (*n* = 125)	−0.186 ± 0.285 (*n* = 49)	0.841
**6 months, mean ± SD**	−0.199 ± 0.199 (*n* = 126)	−0.183 ± 0.229 (*n* = 43)	0.661
**12 months, mean ± SD**	− 0.229 ± 0.190 (*n* = 137)	−0.245 ± 0.211 (*n* = 63)	0.588
**CMT change from Baseline**			
**1 month, mean ± SD**	− 33.95 ± 91.74 (*n* = 151)	−22.54 ± 85.66 (n = 76)	0.367
**3 months, mean ± SD**	−57.50 ± 91.63 (n = 126)	−57.22 ± 104.12 (n = 49)	0.986
**6 months, mean ± SD**	−71.11 ± 93.75 (*n* = 125)	−70.23 ± 113.24 (n = 43)	0.960
**12 months, mean ± SD**	−85.42 ± 109.01 (*n* = 138)	−96.08 ± 101.48 (n = 63)	0.512

*BCVA* Best-corrected visual acuity, *CMT* Central macular thickness

*: Independent t-test

## Discussion

In our study, the rate of PCO in eyes without primary posterior capsulotomy was 13.2%, and Nd:YAG laser was performed in less than half of these eyes (45.0%) for vision-disturbing PCO. Previous studies with a similar setting of combined phaco-vitrectomy reported a PCO rate of 12.5 to 28.4%, [24–26] though the follow-up durations were variable. Long-term evaluation of PCO formation after phaco-vitrectomy is lacking in the literature; however, based on the results of a United Kingdom-based study with a large sample size that showed a gradual increase in PCO incidence (4.7–6.3% at 3 years) and Nd:YAG capsulotomy incidence (2.4–4.4% at 3 years) after cataract surgery, [27] we could infer that visually-significant PCO after phaco-vitrectomy would not be high when patients were followed up for longer periods. Though cautious interpretation is needed as the PCO rate is higher in patients undergoing combined surgery than in patients who undergo cataract surgery alone, [12] the low incidence of visually-significant PCO poses a question as to the necessity of routine primary posterior capsulotomy during phaco-vitrectomy.

Furthermore, there were no differences in the overall surgical outcomes between the groups. In other words, routine posterior capsulotomy during phaco-vitrectomy did not demonstrate any advantage in terms of visual and structural outcomes during the study period.

As for postoperative complications, posterior capsulotomy did not increase the incidence of postoperative inflammation, corneal edema, IOP increase, IOL subluxation or dislocation, postoperative retinal breaks, and ERM recurrence. However, the rate of CME was significantly higher in the capsulotomy (+) group (15.8%) than in the capsulotomy (−) group (6.6%). All eyes with CME in the capsulotomy (−) group received topical eye drops, whereas three eyes (27.3%) in the capsulotomy (+) group were merely observed without medication, depending on the severity of CME. When comparing CME eyes treated with topical eye drops, the CME duration was significantly longer in the capsulotomy (+) group (10 eyes, 3.38 ± 2.39 months) than the capsulotomy (−) group (8 eyes, 1.50 ± 0.71 months) (*p* = 0.030). Therefore, clinicians need to be cautious about the higher rate of CME incidence and longer duration of CME when planning primary posterior capsulotomy.

Postoperative pseudophakic CME is a well-known manifestation of cataract extraction, and it is thought to be associated with multiple factors including inflammatory components and blood-aqueous barrier breakdown [28, 29]. Posterior capsulotomy during phaco-vitrectomy removes the posterior capsule, anterior hyaloid membrane, and the connecting structure between the vitreous gel and posterior capsule, known as Weigert’s ligament, thus making a passage between the posterior chamber and vitreous cavity. Cytokines released by the LECs could be readily transmitted to the vitreous cavity, thereby contributing to the formation of CME [30]. The authors speculate that the prolonged duration of CME after posterior capsulotomy is possibly due to the communication between the vitreous cavity and posterior chamber that remains patent until the posterior capsular circular opening becomes fused with posterior surface of the IOL optic.

In our study, posterior capsulotomy during phaco-vitrectomy and female sex were the independent risk factors for the development of CME after ERM surgery on multivariate logistic analysis. Several previous studies have evaluated risk factors for postoperative CME in the setting of vitrectomy with or without combined cataract surgery for the treatment of idiopathic ERM, [31, 32] however, none have shown a significant sex disparity, in contrast to the results of the present study. We contemplate that the sex disparity of CME occurrence is possibly due to the different sexual hormonal concentrations or anatomical difference of foveal structure [33–35]. Since studies evaluating CME occurrence after phaco-vitrectomy alone are lacking, the findings of the present single study need to be confirmed with future studies.

Meanwhile, other studies have reported the occurrence of postoperative retro-optical opacification with primary posterior capsulotomy, possibly due to the proliferation of LECs on the lens’ posterior surface, [36, 37] though we couldn’t find any case until postoperative 12 months.

This study has several limitations. First, as a retrospective consecutive study, demographic variables such as sex and preoperative baseline CMT were significantly different between the groups. Another drawback of this study was that the surgeries in each group were performed by a different surgeon; and ILM peeling was assisted by either TA or ICG. After the application of TA into the vitreous cavity, the majority of triamcinolone particles would be removed along with the vitreous gel by vitrectomy, but some particles could have been deposited on the retinal surface or remnant vitreous base, thereby protecting against CME occurrence in the capsulotomy (−) group. However, the authors contemplate that the difference between groups would be minimal, because the surgeon of the capsulotomy (+) group also used TA for the visualization of vitreous fibers, [38, 39] and both surgeons meticulously removed the remnant TA to avoid postoperative IOP increase. Lastly, the mean follow-up period of the present study was 11.26 months in total patients, and long-term occurrence of PCO could not be investigated in the study. Further long-term, prospective randomized trials should be conducted to elucidate the results of the present study.

In conclusion, primary posterior capsulotomy during phaco-vitrectomy for idiopathic ERM could eradicate the need for Nd:YAG posterior capsulotomy, but visually-significant PCO that needed Nd:YAG posterior capsulotomy was not common during the postoperative follow-up period (5.9%). The rate of CME occurrence was higher and CME lasted longer in patients who underwent posterior capsulotomy. Primary posterior capsulotomy did not show any advantages in terms of visual and structural surgical outcomes during the study period. Considering the low rate of visually-significant PCO and possible CME after posterior capsulotomy, posterior capsulotomy during phaco-vitrectomy may not be necessary during the vitrectomy procedure.

## Data Availability

The datasets generated during and/or analysed during the current study are available from the corresponding author on reasonable request.
